# Outbreak of Electronic-Cigarette–Associated Acute Lipoid Pneumonia — North Carolina, July–August 2019

**DOI:** 10.15585/mmwr.mm6836e1

**Published:** 2019-09-13

**Authors:** Kevin Davidson, Alison Brancato, Peter Heetderks, Wissam Mansour, Edward Matheis, Myra Nario, Shrinivas Rajagopalan, Bailey Underhill, Jeremy Wininger, Daniel Fox

**Affiliations:** ^1^Pulmonary & Critical Care, WakeMed Hospital, Raleigh, North Carolina; ^2^Department of Pathology, WakeMed Hospital, Raleigh, North Carolina.

*On September 6, 2019, this report was posted as an *MMWR* Early Release on the *MMWR* website (https://www.cdc.gov/mmwr).* Electronic cigarettes (e-cigarettes) produce an aerosol by heating a liquid that usually contains nicotine, flavorings, and other chemicals that users inhale, a behavior commonly referred to as “vaping.” E-cigarettes can also be used to deliver marijuana and other drugs. In recent months, more than 200 possible cases of acute lung injury potentially associated with vaping were reported from 25 states (*1*). During July and August 2019, five patients were identified at two hospitals in North Carolina with acute lung injury potentially associated with e-cigarette use. Patients were adults aged 18–35 years and all experienced several days of worsening dyspnea, nausea, vomiting, abdominal discomfort and fever. All patients demonstrated tachypnea with increased work of breathing on examination, hypoxemia (pulse oximetry <90% on room air), and bilateral lung infiltrates on chest x-ray. All five patients shared a history of recent use of marijuana oils or concentrates in e-cigarettes. All of the products used were electronic vaping pens/e-cigarettes that had refillable chambers or interchangeable cartridges with tetrahydrocannabinol (THC) vaping concentrates or oils, which were all purchased on the street. Three of the patients also used nicotine-containing e-cigarettes, and two of the patients smoked marijuana or conventional cigarettes, although none used other illicit drugs. All five patients were hospitalized for hypoxemic respiratory failure; three required intensive care for acute respiratory distress syndrome, one of whom required intubation and mechanical ventilation. All of the patients survived.

On admission, all patients had an elevated white blood cell count with a neutrophilic predominance and absence of eosinophilia. Initially, all patients were treated empirically with antibiotics (the two-drug combination of ceftriaxone and azithromycin, or a fluoroquinolone) for presumed community-acquired or aspiration pneumonia, but all developed worsening respiratory failure within 48 hours of admission. Blood and sputum cultures were negative for bacterial pathogens; tests for influenza, *Mycoplasma,* and *Legionella* also were negative.

**FIGURE 1:**
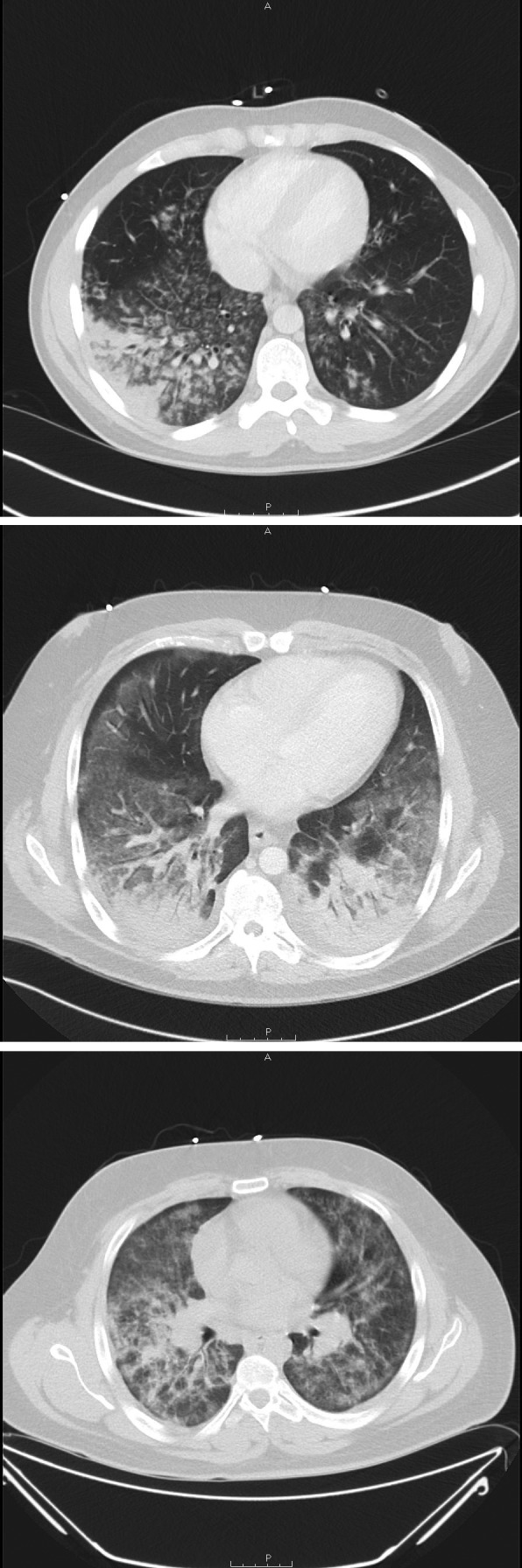
Computerized tomography images showing diffuse lung infiltrates in three patients with e-cigarette–associated severe lung disease — North Carolina, July–August 2019

**FIGURE 2:**
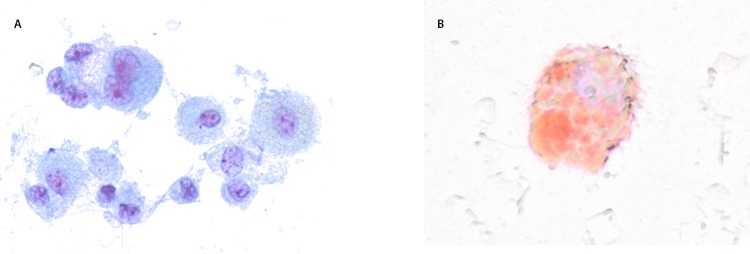
Microscopy of a bronchoalveolar lavage sample (Papanicolaou stain [A]* and oil red O stain [B]^†^) from a patient with acute lung injury associated with vaping — North Carolina, July–August 2019 *Papanicolaou stain demonstrating alveolar macrophages laden with vacuoles. ^†^Oil red O stain showing lipid deposits staining red (400x magnification).

Computed tomography of the chest revealed diffuse basilar-predominant infiltrates with a range of “ground glass” opacities and nodular or “tree-in-bud” infiltrates in all patients (Figure 1). Three patients underwent bronchoscopy with bronchoalveolar lavage on hospital days 3–5, yielding a combination of neutrophils, lymphocytes, and vacuole-laden macrophages, but without evidence for alveolar hemorrhage or eosinophilia (Figure 2). No bronchoscopic lung biopsies were performed. Lavage cytology was stained with oil red O, which confirmed extensive lipid within alveolar macrophages (Figure 2). Based on clinical history, radiography, and laboratory and bronchoscopic diagnostics, a diagnosis of acute exogenous lipoid pneumonia was made for all five patients.

All five patients improved clinically within 24–72 hours after initiation of intravenous methylprednisone (120 mg–500 mg daily). All five patients survived and were discharged home on a taper of oral prednisone.

One potential explanation for acute lipoid pneumonia among these patients is that aerosolized oils inhaled from e-cigarettes deposited within their distal airways and alveoli, inciting a local inflammatory response that impaired vital gas exchange. Lipoid pneumonia has long been described from aspiration of oil into the lungs and has been associated with e-cigarette use in some case reports (*2*–*6*). Symptoms of lipoid pneumonia are often nonspecific with variable chest imaging, which can lead to delayed or missed diagnosis (*6*).

These five cases highlight the importance of awareness of a potential association between use of marijuana oils or concentrates in e-cigarettes and lipoid pneumonia. Diagnosis of lipoid pneumonia among these patients was based on history of using liquids in e-cigarettes that contain sources of lipid, consistent radiologic findings, demonstration of lipid-laden macrophages in respiratory samples, and exclusion of alternative diagnoses. Lipid-laden macrophages are best demonstrated by performing special lipid stains such as oil red O or Sudan staining of cytology from bronchoalveolar lavage (*6*). Further investigation of the specific pathogenesis of acute lung injury and inciting factors are warranted to determine whether other cases in the ongoing multistate outbreak (*1*) bear the same features as the cases described in this report. Patients with lipoid pneumonia might improve on corticosteroids; however, the optimal treatment regimen and duration, as well as the long-term effects of this lung injury, are uncertain (*6*).
